# Incidence and Risk Factors of Refractive Error in Children in Spain: CISViT Project

**DOI:** 10.1007/s44402-026-00086-4

**Published:** 2026-05-05

**Authors:** Alba Galdón, Laura Guisasola, Mariam El Gharbi, Valldeflors Vinuela-Navarro, Joan Pérez-Corral, Núria Vila-Vidal

**Affiliations:** 1 Visió Optometria i Salut research group, Department of Optics and Optometry, Universitat Politècnica de Catalunya Terrassa, Barcelona, Spain; 2 Centre Universitari de la Visió, Universitat Politècnica de Catalunya Terrassa, Barcelona, Spain

**Keywords:** Children, Myopia, Myopic risk factors, Refractive error

## Abstract

**Background:**

The incidence of refractive errors (RE), particularly myopia, has increased in recent years, presenting significant geographic variations. This prospective longitudinal cohort study aims to assess the incidence of RE in a school-based cohort in northeastern Spain and identify associated risk factors.

**Methods:**

The study followed up 1189 children with an ~1-year interval (mean age: 8.74 years at visit 1 and 10.00 years at visit 2) from a school-based cohort in northeast Spain (2021–2024). RE was measured using an autorefractometer and retinoscopy without cycloplegia. A preliminary questionnaire was used to collect data on potential risk factors such as visual habits and socioeconomic status.

**Results:**

The spherical equivalent (SE) at the initial visit was +0.29 ± 0.03 D, decreasing to +0.14 ± 0.04 D at follow-up. The prevalence of myopia increased from 12.3% at baseline to 17.1% at follow-up. A total of 56 incident cases were identified, corresponding to a cumulative incidence of 5.4%. In multivariable logistic regression, baseline SE ≤ +1.00 D was associated with a clinically significant myopic shift (odds ratio (OR) = 1.67, 95% CI 1.29–2.16; *p* < 0.001). Parental myopia was strongly associated with the outcome (maternal: OR = 23.36, 95% CI 8.33–65.49; *p* < 0.001; paternal: OR = 4.25, 95% CI 1.74–10.38; *p* < 0.001). Lower outdoor exposure was also associated with higher odds of a clinically significant myopic shift (low vs. high: OR = 9.33, 95% CI 5.89–14.79; *p* < 0.001). Parental education was not significantly associated, whereas parental unemployment was associated with higher odds of myopic shift in the fully adjusted model.

**Conclusions:**

The incidence of myopia and progression of RE (change in SE) in this school-based cohort of northeastern Spanish schoolchildren was significant over 1 year. Factors such as paternal/maternal myopia, reduced outdoor time and both paternal and maternal unemployment were associated with a higher risk of a clinically significant myopic shift (ΔSE ≤ −0.50 D).

Key Points

- This longitudinal study provides new data on myopia in southern Europe, where such data are scarce, showing that one-year myopia incidence in children aged 8–10 years is substantial and warrants preventive action.
- Parental myopia and reduced time outdoors are the strongest risk factors associated with a clinically significant myopic shift, highlighting clear targets for clinical and public health action.
- Socioeconomic factors, particularly parental unemployment, are associated with greater myopic progression, suggesting that family circumstances influence refractive development beyond individual behaviours.

## Introduction

Refractive errors (RE), such as myopia, hyperopia and astigmatism, are common visual impairments in the paediatric population that affect visual acuity (VA) at both near and far distances [1]. These conditions can interfere significantly with children’s academic and social development [2–4]. Given their impact on school performance and quality of life, early detection and correction are essential [5]. Beyond their prevalence, the severity of RE is also a critical factor, as it has been demonstrated that visual deterioration associated with myopia worsens as its magnitude increases [6].

Hyperopia has been linked with reading difficulties, suggesting that poor vision may affect the acquisition of fundamental academic skills [7–10]. In addition to its impact on learning, RE is associated with various ocular conditions. Significant hyperopia (≥2 D) is linked with an increased risk of developing strabismus and amblyopia, while uncorrected myopia can lead to symptoms of visual fatigue and ocular discomfort [11, 12]. Notably, higher levels of myopia present a 10–40 fold increased risk of developing severe ocular pathologies compared with lower levels [13–15]. Therefore, identifying myopic children will allow for the implementation of refractive and pharmacological interventions to slow myopic progression [16].

Over the last few decades, a steady increase in childhood myopia has been reported worldwide. Prevalence is particularly high in East Asian populations, where it exceeds 80% amongst adolescents, but it is also rising in many Western countries [17]. Projections estimate that by 2050, nearly half of the world’s population will be myopic, with almost one billion individuals affected by high myopia [18]. The age of onset is a key determinant of long-term prognosis: children who become myopic at a younger age tend to show faster progression and are more likely to reach high myopia in later adolescence [19]. In this context, the years corresponding to late primary school represent a critical developmental window for detecting early myopic changes and implementing preventive or control strategies. In addition to the age of onset, parental myopia is one of the strongest non-modifiable predictors of childhood myopia [20]. Myopic progression is driven mainly by axial elongation, which underlies the long-term risk of ocular complications [20]. Also, the International Myopia Institute (IMI) defines pre-myopia as a risk state in which children are not yet myopic but have a high likelihood of developing myopia [21]. These considerations support focusing on ages 8–10 years, when many children transition from low hyperopia/pre-myopia to manifest myopia.

Environmental and behavioural factors influence myopia development. Prolonged near-work activities, such as reading and screen use, are risk factors, especially in young children [22, 23]. The Sydney Adolescent Vascular and Eye Study (SAVES), conducted in Australian schoolchildren, found a significant association in 6-year-olds but not in older children, suggesting that early near-work exposure may contribute to myopia onset [24]. Additionally, shorter reading distances and extensive study periods increase both onset and progression [25, 26]. Along with these modifiable exposures, family history is widely recognised as an important determinant of myopia risk in childhood, and baseline refractive status in the early school years may help identify children at a higher likelihood of developing myopia during follow-up [27–29].

Conversely, outdoor time is one of the primary protective factors against myopia. Studies such as SAVES and *Avon Longitudinal Study of Parents and Children* (ALSPAC, England) have demonstrated that children who develop myopia spend fewer hours outdoors than those who do not [24, 30]. Clinical trials have shown that increasing daily exposure to outdoor light can reduce the incidence and progression of myopia by up to 30.4% [22]. This effect appears to be mediated by the release of retinal dopamine, which regulates ocular growth [31]. Other factors, such as exposure to ultraviolet light and vitamin D [32], may also play a role, although findings remain inconsistent [33]. Despite this, most specialists recommend increasing exposure to natural light as an effective preventive strategy [34].

Socioeconomic status (SES) influences the prevalence of myopia, but its impact appears complex and context dependent. In India, children with higher SES and those attending private schools exhibit a higher prevalence of myopia, which may be related to greater academic demands and increased near-work activities [35]. In the Netherlands, a higher risk of myopia has been observed in children from lower SES backgrounds and non-European origin [36]. Importantly, this relationship should be interpreted cautiously, as SES likely acts as a proxy for clusters of environmental and behavioural exposures rather than as a direct cause of myopia. Evidence from disadvantaged or Indigenous populations in different settings further suggests that low SES is not uniformly associated with higher myopia prevalence; in some communities, myopia remains relatively low while uncorrected refractive error and limited access to eye-care services may represent a greater concern [37, 38]. These findings underscore the need to interpret socioeconomic associations in light of educational intensity, lifestyle patterns, cultural context and access to care.

Evidence from European and Mediterranean countries suggests that childhood myopia is increasing, although prevalence and progression rates vary considerably by region and age. In the United Kingdom, the *Northern Ireland Childhood Errors of Refraction* (NICER) study documented a more than twofold increase in myopia prevalence among white children over the past half-century, with incidence peaking in early adolescence [39]. Similarly, the Israel Refraction, Environment, and Devices (iREAD) study in Israel reported significant 12-month refractive shifts, particularly amongst Ultra-Orthodox boys, highlighting how educational intensity and parental myopia, rather than screen time alone, drive progression [40]. Recent meta-analyses estimate a pooled myopia prevalence of 7.15% among European children, although national estimates range widely, from 19% in French 9‑year‑olds to near 0% in younger Danish cohorts [41]. In Spain, serial cross‑sectional surveys showed a steady rise in myopia prevalence among 5‑ to 7-year-olds from 16.8% in 2016 to 20.4% in 2019, with lifestyle and family history emerging as key risk factors [42]. While these studies confirm a growing burden, most remain cross‑sectional. There is still limited longitudinal evidence from Southern Europe, particularly from Spain, on how refractive status evolves during the critical late primary‑school years (ages 8–10 years) and which behavioural and socioeconomic factors are most strongly associated with the onset and short‑term progression of myopia in this age range.

In this context, the present study forms part of the Cohort Infantil de Salut Visual de Terrassa (CISViT) project and uses a prospective longitudinal cohort of 1189 schoolchildren from Terrassa, northeastern Spain, aged 8–10 years, examined at baseline and after 1 year. The primary objective was to estimate the 1-year incidence and progression of myopia in this cohort and to identify associated risk factors, focusing on baseline refractive status, age, visual habits (near-work activities and outdoor exposure) and indicators of SES. As secondary objectives, the overall distribution and evolution of RE (myopia, emmetropia and hyperopia) is described over the follow-up period. This longitudinal design allows the investigation to go beyond simple prevalence estimates and to characterise which children are at greatest risk of developing or worsening myopia during the late primary-school years.

## Methodology

### Study Participants

This prospective longitudinal cohort study on RE included 1189 children aged 8–10 years from 16 primary schools in northeastern Spain. A two-stage cluster sampling design was used: public and publicly funded private schools were randomly selected from official lists and all children in the grades corresponding to 8–10 years of age in participating schools were invited to take part. Participants underwent a baseline eye examination and were re-examined ~12–15 months later (follow-up visit) to assess refractive change over time.

Information letters and consent forms were distributed through the schools. Participation required written informed consent from parents or legal guardians and assent from the child (Appendix 1). Examinations were performed at the Centre Universitari de la Visió (CUV) by licensed optometrists from the Faculty of Optics and Optometry of Terrassa (Universitat Politècnica de Catalunya). All examinations were provided free of charge.

Children were eligible if they were 8–10 years of age, enroled in participating schools and had consent. Children with complete RE measurements at both visits were included. Exclusion criteria were limited to prior ocular surgery, ocular pathology affecting refraction (e.g., congenital cataract, corneal dystrophy, glaucoma or uveitis) or systemic conditions known to severely affect visual development. Conditions commonly associated with refractive development (e.g., amblyopia, strabismus, preterm birth or binocular vision abnormalities) were not used as exclusion criteria in order to reflect the general school population.

### Sample Size

The sample size was calculated using EpiData version 3.1 (epidata.dk) for estimating the 1-year incidence of myopia in this cohort. An expected cumulative incidence of 5% in this age range, an absolute precision of 2% and a 95% confidence level were assumed. Under a simple random sampling design, the required sample size was 456 children. Because a school-based cluster sampling plan was used, a design effect of 2.0 was applied, resulting in a minimum required sample of 912 children completing follow-up. Assuming an 80% response rate (i.e., up to 20% loss to follow-up or non-response), the target baseline sample was 1140 children. Ultimately, 1189 children were recruited at baseline and completed the 1-year follow-up with valid refractive measurements, thus exceeding the minimum required sample size.

### Experimental Procedure

The study was conducted between September 2021 and September 2024, with each child examined at two time points (the initial visit and 1-year follow-up). Before each evaluation, informed consent from parents or guardians was confirmed and each child received a detailed explanation of the procedures.

### Refractive Error Assessment

Refractive error (RE) was determined under non-cycloplegic, open-field conditions using an autorefractometer Shin-Nippon NVision-K 5001 (rexxam.co.jp/eye-care). Children were instructed to fixate a distant target at 3 m and at least three readings were obtained for each eye; only measurements with an appropriate quality signal were accepted and the mean value was used for analysis. To reduce accommodative artefacts and verify the autorefractor results, static retinoscopy was subsequently performed by experienced optometrists in a dimly lit room. A standard fogging technique was used to relax accommodation: the examination was initiated with plus lenses (+1.50 to +2.00 D) and the power was reduced in 0.25 D steps until neutrality was reached. Myopic reversal was checked by adding −0.25 to −0.50 D lenses to confirm the endpoint.

The spherical equivalent (SE) was calculated as the spherical power plus half of the cylindrical power. If the difference in SE between the autorefractor and retinoscopy exceeded 0.50 D in either eye, then the measurements were repeated. When a persistent discrepancy ≥0.50 D remained after repetition, the retinoscopy value was taken as the final refraction for that eye. Differences <0.50 D were considered clinically acceptable and the autorefractor value was retained. For all analyses, only data from the right eye were used. Preliminary analyses in the CISViT cohort, using Pearson’s correlation coefficient, showed a very high correlation in SE between the right and left eyes (*r* = 0.84, 95% CI [0.82, 0.86], *p* < 0.001).

### Definition and classification of refractive error and progression

Refractive status was classified based on the SE of the right eye. Myopia was defined as SE ≤ −0.50 D, in line with the IMI consensus definition for epidemiological studies [21]. Emmetropia was defined as −0.50 D < SE < +1.00 D and hyperopia as SE ≥ +1.00 D [41, 42]. Because refraction was measured without cycloplegia, a conservative cut-off for hyperopia (+1.00 D) was used to reduce misclassification of latent hyperopia as emmetropia due to accommodative effects [21].

Myopia incidence was defined at the child level as the proportion of participants who were non-myopic at baseline (SE > −0.50 D) and became myopic (SE ≤ −0.50 D) at follow-up. Refractive change was quantified as the difference in SE between visits (ΔSE = SE follow-up−SE baseline). For regression analyses, the primary outcome was clinically significant myopic shift, defined as ΔSE ≤ −0.50 D over follow-up. This threshold was selected to exceed the expected repeatability limits of non-cycloplegic refraction and to represent a clinically meaningful magnitude of change over ~1 year in school-aged children [43].

### Questionnaire on visual behaviours

A structured paper questionnaire was distributed to parents/guardians before the clinical examination. This instrument was specifically developed for the CISViT project, drawing from items used in previous studies on children’s visual habits, although it has not undergone formal psychometric validation or test–retest reliability assessment (Appendix 1). In addition to lifestyle exposures, the questionnaire collected information on family history of myopia, including maternal and paternal myopia (self-reported by parents/guardians). Parents reported their child’s average daily time spent on near-vision activities (reading, writing/homework, digital device use) and outdoor activities for weekdays. Weighted average daily durations were computed from these reports. For analysis, near-work and outdoor time were dichotomised at 2 h/day (high exposure ≥ 2 h/day; low exposure < 2 h/day), consistent with thresholds commonly used in epidemiological myopia research.

SES was assessed using parental education and employment status reported in the questionnaire. For each child, the highest educational level attained by either parent was classified into two categories (low level: primary or less; high level: secondary, university) and the employment status of each parent was recorded as employed or unemployed.

### Participant Flow

**Fig. 1:**
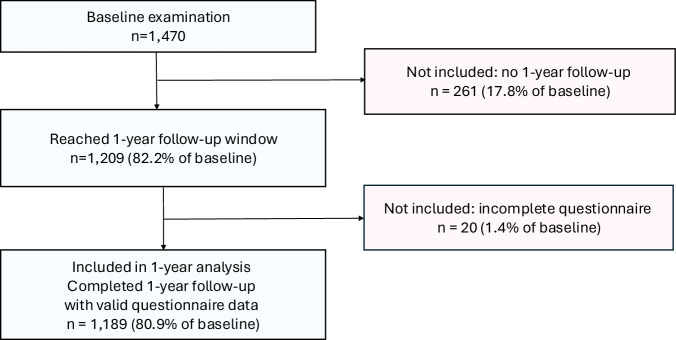
Flow of participants through the Cohort Infantil de Salut Visual de Terrassa (CISViT) study.

Figure 1 shows the flow of participants through the study. A total of 1470 children completed the baseline examination. Of these, 1209 children (82.2% of baseline) had reached the 1-year follow-up window at the time of analysis; the remaining 261 (17.8%) did not attend the 1-year follow-up visit. Among the 1209 eligible children, 1189 (80.9% of baseline) completed the 1-year follow-up examination with valid refractive and questionnaire data and were included in the longitudinal analyses, whereas 20 children (1.4% of baseline) were excluded because of incomplete questionnaire data.

### Statistical Analysis

Descriptive statistics were calculated at baseline and follow-up. Normality of the SE was assessed using the Kolmogorov–Smirnov test and visual inspection. Because the SE was not normally distributed, sex differences in SE at each visit were evaluated using the Mann–Whitney *U* test. Refractive error categories (myopia, emmetropia, hyperopia) were summarised as proportions with 95% confidence intervals (CI) and compared by sex using Pearson’s *χ*² tests. Myopia incidence was defined as a transition from baseline non-myopia (hyperopia or emmetropia) to myopia at follow-up, and was calculated using the baseline non-myopic denominator; sex differences in incidence and refractive-category transitions were assessed using *χ*² tests. All tests were two-sided and *p* < 0.05 was considered statistically significant.

For the main risk-factor analysis, multivariable binary logistic regression was used to identify factors associated with clinically significant myopic shift, defined as ΔSE ≤ −0.50 D in the right-eye SE over follow-up. Because the dependent variable is based on within-child change between visits, the regression models directly address longitudinal change. Given non-cycloplegic refraction, baseline SE was included a priori in all models. Predictors were selected a priori based on biological plausibility and prior evidence and entered using a hierarchical modelling strategy (no stepwise procedures): Model 1 (age, sex, baseline SE, parental/maternal myopia), Model 2 (Model 1 + outdoor time and near-work exposure) and Model 3 (Model 2 + parental education and parental employment). Results are presented as adjusted odds ratios (aORs) with 95% CI and Wald *p*-values.

Multicollinearity among behavioural and socioeconomic predictors was evaluated using tolerance and variance inflation factors (VIF) from auxiliary regression models. Exploratory interaction terms (age × sex and sex × behavioural factors) were examined; they were not retained because they were not statistically significant and did not improve model fit; therefore, only main-effect models are presented. Analyses were performed using IBM SPSS (ibm.com), with *p* < 0.05 considered statistically significant.

## Results

A total of 1189 schoolchildren participated at baseline, including 625 boys (52.6%) and 564 girls (47.4%). The mean age at baseline was 8.74 ± 0.53 years. The same cohort was re-evaluated at follow-up (~1 year later), when the mean age was 10.00 ± 0.68 years.

### Spherical Equivalent of the Refractive Error

**Fig. 2:**
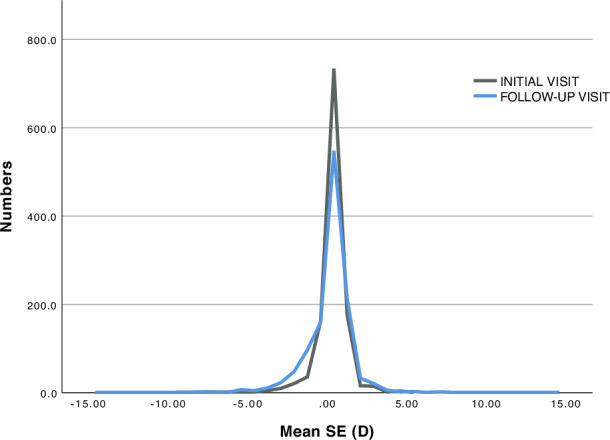
Distribution of mean spherical equivalent (SE) in children at the initial and follow-up visits.

The mean SE was analysed in both visits. At the initial visit, the mean SE was +0.29 ± 0.03 D, while at the 1-year follow-up, the mean SE was +0.14 ± 0.04 D (Fig. 2). Both sexes showed identical mean SE at follow-up (+0.14 ± 0.06 D). At baseline, mean SE was slightly higher in boys than in girls (+0.31 D vs. +0.27 D). Mean SE was compared between boys and girls using the Mann–Whitney *U* test due to non-normality, showing no significant sex difference at baseline (*U* = 175,89; *Z* = −0.06; *p* = 0.95).

### Prevalence of Myopia, Hyperopia and Emmetropia

At the initial visit, most children presented with emmetropia, followed by lower proportions of hyperopia and myopia. At follow-up, a general shift in refractive error toward more negative values was observed, with an increase in the prevalence of myopia. Although girls showed a slightly greater increase in myopia compared with boys, sex differences were not statistically significant at either the initial or the follow-up visit (Table 1).

**Table 1:** Prevalence of myopia, hyperopia and emmetropia by sex at the initial and follow-up visits.

Refractive error	Initial visit			Follow-up visit		
	Sex		Total	Sex		Total
	Male	Female		Male	Female	
Myopia *n* (%) 95% CI	75 (12.0) [9.5, 14.6]	71 (12.6) [9.9, 15.3]	146 (12.3) [10.5, 14.1]	95 (15.2) [12.4, 18.0]	107 (19.0) [15.9, 22.1]	202 (17.1) [14.9, 19.1]
Emmetropia *n* (%) 95% CI	444 (71.0) [67.5, 74.5]	408 (72.3) [68.6, 76.0]	852 (71.7) [69.1, 74.3]	438 (70.1) [66.5, 73.7]	375 (66.5) [62.6, 70.4]	813 (68.3) [65.7, 71.1]
Hyperopia *n* (%) 95% CI	106 (17.0) [14.0, 20.0]	85 (15.1) [12.3, 17.9]	191 (16.0) [14.0, 18.2]	92 (14.7) [11.9, 17.5]	82 (14.5) [11.7, 17.3]	174 (14.6) [12.6, 16.6]
Total *n*	625	564	1189	625	564	1189

Table 1 illustrates the distribution of RE (myopia, emmetropia and hyperopia) at the initial and follow-up visits, stratified by sex. The table highlights temporal changes in refractive status, showing a slight increase in the proportion of subjects with myopia and a corresponding decrease in emmetropia in both males and females. Hyperopia prevalence remained relatively stable throughout the observation period. No significant sex differences were observed in the distribution of refractive error categories at baseline or follow-up (baseline *χ*²(2) = 0.81, *p* = 0.67; follow-up *χ*²(2) = 3.05, *p* = 0.22), although girls showed a small, non-significant tendency toward higher myopia prevalence at both visits.

### Categorisation of Refractive Error Changes

During the follow-up (≈1 year), 56 incident myopia cases occurred among the 1043 children who were non-myopic at baseline, yielding a cumulative incidence of 5.4%. Incident myopia was more frequent in girls than in boys (36/493 [7.3%] vs. 20/550 [3.6%]); this sex difference was statistically significant (Pearson’s *χ*²(1) = 6.88, *p* = 0.009).

**Fig. 3:**
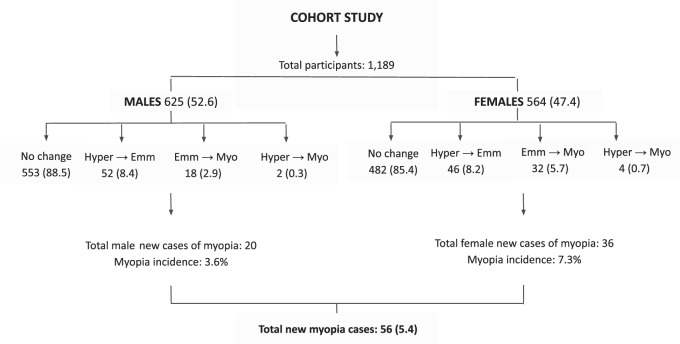
Flowchart of refractive error category transitions during follow-up by sex. Emm emmetropia, Hyper hyperopia, Myo myopia.

Figure 3 summarises the right-eye refractive error category transitions during follow-up among the 1189 children with complete data, stratified by sex (boys: *n* = 625; girls: *n* = 564). Transitions were classified as no change, hyperopia → emmetropia (physiological shift), emmetropia → myopia and hyperopia → myopia. Overall, 154 children (12.9%) changed refractive category; 56 children transitioned from non-myopia to myopia (incident myopia; 56/1043 [5.4%] of baseline non-myopic children), accounting for the increase in myopia prevalence from 146 at baseline to 202 at follow-up. The distribution of refractive category transitions did not differ between boys and girls (*χ*²(3) = 2.76, *p* = 0.43).

### Degree of Myopia Progression

Changes in SE were analysed in the same cohort of 1189 children. Overall, 75.4% (896/1189) showed no clinically significant myopic shift (ΔSE > −0.50 D), while 24.6% (293/1189) showed a clinically significant myopic shift (ΔSE ≤ −0.50 D). When stratified by sex, these proportions did not differ significantly (*p* = 0.13).

Table 2 presents the change in SE (ΔSE) in the right eye by baseline refractive category and age group. Hyperopic children showed the greatest myopic shift, particularly in the older age group: mean ΔSE was −0.04 ± 1.72 D in the 8–<9-year age group and −0.42 ± 1.43 D in the 9–<10-year age group. A clinically significant myopic shift (ΔSE ≤ −0.50 D) occurred in 44.6% and 61.1% of these children, respectively.

**Table 2:** Change in spherical equivalent (ΔSE, right eye) during follow-up by baseline refractive status and age group.

Baseline refractive status	Age group	*n*	ΔSE (D), mean ± SD	ΔSE ≤ − 0.50 D, *n*/*N* (%)
Hyperopic	8–<9 years	101	−0.04 ± 1.72	45/101 (44.6)
	9–<10 years	90	−0.42 ± 1.43	55/90 (61.1)
Emmetropic	8–<9 years	412	0.08 ± 0.90	100/412 (24.3)
	9–<10 years	440	0.00 ± 1.22	59/440 (13.4)
Myopic	8–<9 years	43	0.00 ± 1.87	8/43 (18.6)
	9–<10 years	103	−0.17 ± 1.45	26/103 (25.2)

Age categories were defined as closed-open intervals [8–<9 years) and [9–<10 years). Children exactly 9.0 years of age were included in the 9–<10 years group.

Amongst emmetropic children, mean ΔSE was 0.08 ± 0.90 D in the 8–<9-year age group and 0.00 ± 1.22 D in the 9–<10-year age group; the proportion with ΔSE ≤ −0.50 D was 24.3% and 13.4% in the younger and older group, respectively. In contrast, children who were myopic at baseline showed smaller average changes (0.00 ± 1.87 D in the 8–<9-year-olds and −0.17 ± 1.45 D in the 9–<10-year-olds), with 18.6% and 25.2% showing an additional myopic shift of ΔSE ≤ −0.50 D, respectively. Overall, refractive change towards myopia was most pronounced among children who were hyperopic or emmetropic at baseline.

### Analysis of Factors Related to Changes in Refractive Error

Multivariable binary logistic regression was used to identify factors associated with a clinically significant myopic shift, defined as ΔSE ≤ −0.50 D in right-eye SE at follow-up. In the fully adjusted model (Table 3), baseline non-cycloplegic SE was independently associated with the outcome (odds ratio (OR) = 1.67, 95% CI 1.29–2.16, *p* < 0.001). Parental myopia was a strong predictor: maternal myopia (OR = 23.36, 95% CI 8.33–65.49, *p* < 0.001) and paternal myopia (OR = 4.25, 95% CI 1.74–10.38, *p* < 0.001) were associated with higher odds of clinically significant myopic shift. Sex was not associated with the outcome (*p* = 0.44).

**Table 3:** Multivariable logistic regression analysis of factors associated with clinically significant myopic shift (ΔSE ≤ −0.50 D).

Risk factor (response rate %)	ΔSE [≤−0.50 D] (*n*)/total (*N*)	(%)	OR (95% CI)	*p* value
*Sex*				
Boys	145/625	23.2	Ref	
Girls	148/564	26.2	1.18 (0.77–1.82)	*p* = 0.44
*Age reference category*				
8–9 years	183/849	21.6	Ref	
10–11 years	110/340	32.4	1.26 (1.10–1.44)	*p* < 0.001*
*Initial refractive error*				
SE > 1.00 D Initial Visit	90/191	47.1	Ref	
SE ≤ 1.00 D Initial Visit	203/998	20.3	1.67 (1.29–2.16)	*p* < 0.001*
*Myopic father*				
No	189/1000	18.9	Ref	
Yes	104/189	55.0	4.25 (1.74–10.38)	*p* < 0.001*
*Myopic mother*				
No	139/1010	13.8	Ref	
Yes	154/179	86.0	23.36 (8.33–65.49)	*p* < 0.001*
*Educational level father*				
High educational level	233/746	31.2	Ref	
Low educational level	60/443	13.5	0.78 (0.43–1.41)	*p* = 0.41
*Educational level mother*				
High educational level	243/761	31.9	Ref	
Low educational level	50/428	11.7	1.77 (0.97–3.23)	*p* = 0.06
*SES work father*				
Employed	203/857	23.7	Ref	
Unemployed	90/332	27.1	2.36 (1.44–3.86)	*p* < 0.001*
*SES work mother*				
Employed	193/625	30.9	Ref	
Unemployed	100/564	17.7	2.56 (1.58–4.14)	*p* < 0.001*
*Near-vision activities*				
Low exposure	203/694	29.3	Ref	
High exposure	90/494	18.2	1.48 (0.95–2.31)	*p* = 0.08
*Outdoor exposure time*				
High exposure	52/726	7.2	Ref	
Low exposure	241/463	52.1	9.33 (5.89–14.79)	*p* < 0.001*

*CI* confidence interval, *OR* odds ratio, *SE* spherical equivalent, *SES* socioeconomic status.

Significance level:*p < 0.05.

Among behavioural and socioeconomic factors, lower outdoor exposure was strongly associated with higher odds of a clinically significant myopic shift (low vs. high outdoor time: OR = 9.33, 95% CI 5.89–14.79, *p* < 0.001). Near-work exposure showed a non-significant trend (OR = 1.48, 95% CI 0.95–2.31, *p* = 0.08). Socioeconomic indicators showed limited and mixed associations: parental education was not significantly associated (father *p* = 0.41; mother *p* = 0.06), whereas parental employment status was associated with the outcome (father *p* < 0.001; mother *p* < 0.001) (Table 3). Given the use of non-cycloplegic refraction, these findings should be interpreted cautiously, particularly when considering small changes in SE.

## Discussion

This longitudinal study describes refractive changes and myopia incidence in a school-based cohort from Terrassa, northeastern Spain, examined in late primary school (baseline age 8–9 years) and reassessed ~1 year later. In addition, biological, behavioural and socioeconomic correlates of a clinically meaningful myopic shift (ΔSE ≤ −0.50 D) were evaluated.

At baseline, emmetropia was the most prevalent refractive error (71.7%), followed by hyperopia (16.0%) and myopia (12.3%); the latter being the least common. Myopia prevalence increased to 17.1% at the 1-year follow-up. The observed baseline myopia prevalence falls within the range reported in other school-aged cohorts, including Bosnia 17.3% (children aged 7–16 years) [44], Australia 14.7% (12 years) [45], urban public schoolchildren in the USA 9.4% (grades K–5; 5–11 years) [46] and Ireland 22.8% (12–13 years) [47]. In contrast, it remains markedly lower than estimates reported in East Asian settings (e.g., 63.1–76.5%) [48–50] and Canada 28.9% (1(13–15 years) [51]. Other studies reported more moderate prevalences, such as in Pakistan 14.02% (5–10 years) [52], while a report from Saudi Arabia noted comparatively low myopia prevalence (4.5% in 12–13 years) [53]. However, these studies were not strictly age-matched to the current 8–10-year-old cohort and differed in refraction protocols (cycloplegic vs. non-cycloplegic) and myopia definitions, which may influence prevalence estimates. Therefore, cross-study comparisons should be interpreted cautiously.

These disparities likely reflect differences in environmental exposures, geographical context and ethnicity, which contribute to distinct prevalence patterns. In East Asia, the combination of intense educational demands and limited outdoor time has been consistently linked with higher myopia prevalence, whereas a more intermediate prevalence in parts of Europe and in the present study may reflect a different balance of visual habits and lifestyle factors. Notably, low prevalence estimates have been reported in Poland (5.7–8.3% in 9–13 years; boys-girls) [54] and India (4.4% in 7–15 years) [55] and a relatively low prevalence has also been observed in Norwegian adolescents (12.7% in 16–19 years) [56]. Together, these findings underscore how myopia prevalence varies widely across settings and should be interpreted in light of age, study design, local behavioural and educational patterns.

No significant differences were observed in refractive error distribution by sex at baseline or at follow-up. This finding suggests that, at least within this age group, sex is not a major determinant of refractive error distribution, consistent with prior European studies [2, 57, 58]. However, when incident myopia was calculated using the appropriate baseline non-myopic denominator, girls showed a higher 1-year cumulative incidence than boys (7.3% vs. 3.6%); a difference that reached statistical significance (*χ*²(1) = 6.88, *p* = 0.009). This sex difference in incident myopia has been reported in other cohorts and may become more evident at older ages, warranting confirmation in longer-term longitudinal studies [59, 60].

The majority of participants (75.4%) maintained stable refractive status. Nevertheless, 24.6% exhibited a clinically significant myopic shift (ΔSE ≤ −0.50 D). This threshold was used as the primary outcome because it exceeds the expected repeatability limits of non-cycloplegic refraction and represents a clinically meaningful magnitude of change over ~1 year. Importantly, this outcome captures refractive change across a threshold and includes children who may not meet the myopia definition at follow-up; therefore, the identified associations should be interpreted as correlates of refractive change (myopic shift) rather than myopia progression as a disease process. Though a minority, this subgroup represents a critical at-risk population, given the established association between higher myopia and long-term ocular complications [61–64].

At baseline, the mean SE was +0.29 D, declining to +0.14 D after 1 year. This shift reflects a tendency towards myopisation within the cohort, corroborating previous research [65]. This progression rate (–0.15 D) mirrors findings from a London, UK-based study [66], but is substantially lower than rates reported in East Asia. For instance, Saw et al. [67]. documented an annual progression of –0.47 D among Singaporean children 7–12 years of age, while the Shunyi District study (China) reported an annual change of –0.42 D [68]. Because refraction was non-cycloplegic and axial length was not measured, small mean changes should be interpreted cautiously; refractive change cannot be directly attributed to axial elongation in the current study.

The myopia incidence rate was 5.4%, lower than findings from Asian populations such as Hong Kong (9% at ages 7–8 years, rising to 14–18% at 11–12 years of age) [69, 70], further underscoring geographical and ethnic disparities.

Multivariate analysis yielded results consistent with prior research. Regarding SES, parental unemployment, particularly maternal, was associated with greater refractive error progression (more negative change in SE) [71], possibly reflecting reduced access to preventive measures (e.g., outdoor activities) [72]. Although parental education level showed a similar trend, its effect was not significant, suggesting that employment status may capture a child’s sociocultural environment better, as posited by a Rotterdam study [36]. These socioeconomic associations should be interpreted cautiously: effect sizes were not consistent across all SES indicators and SES likely acts as a proxy for clusters of behavioural and environmental exposures rather than a direct cause.

Parental myopia was assessed by self-report without clinical verification or information on severity, which may introduce misclassification and limit the precision of effect-size estimates. In addition, some observed associations (e.g., for parental myopia and outdoor exposure) were relatively large and may partly reflect residual confounding and correlated exposures or proxy influences (e.g., shared family behaviours and socioeconomic context), particularly given non-cycloplegic refraction and the absence of biometric measurements. Therefore, these estimates should be interpreted cautiously and should not be taken as isolated causal effects.

Contrary to the current findings, several studies have reported a distinct pattern. For instance, in communities where formal schooling is limited (e.g., rural or indigenous populations) [73, 74], myopia is virtually absent. Conversely, in societies with rigorous educational systems [75], childhood myopia rates escalate sharply. This supports the view that contextual factors (educational intensity, lifestyle and family history) may shape myopia risk through multiple indirect pathways.

A key finding was the association of outdoor exposure: children spending ≥2 h daily outdoors exhibited less negative refractive change (smaller myopic shift in SE) and reduced odds of a clinically significant myopic shift. This is consistent with mounting evidence advocating natural light exposure as a primary strategy for myopia prevention [76–79]. In contrast, near-work activities showed no significant association, aligning with current literature where such links remain weak and inconsistent [20, 80]. Given the questionnaire-based, coarsely categorised and dichotomised measurement of near work, this null finding should not be overinterpreted; limited exposure resolution may have reduced the ability to detect dose–response relationships.

### Limitations

Refractive error was measured without cycloplegia, which may introduce accommodative bias and substantial measurement variability. This limitation is reflected in the relatively large SDs observed for ΔSE (Table 2), which are higher than typically reported in paediatric studies using cycloplegic refraction and reduce confidence in the magnitude of refractive error change when analysed as a continuous outcome. Accordingly, small mean changes in SE should be interpreted cautiously. To mitigate this, refraction was obtained under open-field conditions with repeated measurements and verified by static retinoscopy using a fogging technique; nevertheless, measurement error and some misclassification around refractive cut-offs remain possible. To improve clinical interpretability and reduce sensitivity to random measurement noise, the main analyses focused on a clinically meaningful threshold (ΔSE ≤ −0.50 D); however, this approach mitigates but does not eliminate the limitation related to measurement variability.

Axial length was not measured, so refractive changes cannot be directly attributed to axial elongation. Follow-up was relatively short (≈1 year), limiting inference on longer-term trajectories and reducing stability for outcomes with relatively few events. Although the analysis adjusted for major covariates (baseline SE, age, sex, parental myopia and key behavioural/SES indicators), residual confounding cannot be excluded.

Behavioural exposures (outdoor time and near-work) and parental myopia/SES indicators were parent-reported using a study-specific questionnaire and broad categories (e.g., ≥2 h/day), which may lead to recall error and non-differential misclassification and could attenuate associations. In particular, parental myopia was assessed by self-report without clinical verification or information on severity/onset, which may introduce misclassification and limit the precision of the estimated effect sizes; therefore, the observed associations should be interpreted cautiously as reflecting family-history/proxy influences rather than precise effect-size estimates.

Finally, this study was based in a single city (Terrassa) in northeastern Spain and does not constitute a nationally representative sample. Therefore, the findings may not be generalisable beyond similar urban settings in this region. Seasonality and school-calendar timing may have influenced outdoor and near-work behaviours and potential post-COVID pandemic behavioural changes were not quantified directly, although data collection occurred when routine schooling had resumed in Spain.

## Conclusion

This longitudinal study highlights the moderate prevalence and incidence of myopia in schoolchildren aged 8–10 years from Terrassa, northeastern Spain, with a clear trend toward myopisation over the course of 1 year. Although most children maintained a stable refractive status, a relevant proportion showed a clinically significant myopic shift (ΔSE ≤ −0.50 D)/refractive error progression, underscoring the importance of early detection and intervention.

In line with current literature, the results indicate that refractive error progression and myopia incidence were associated with modifiable environmental factors, particularly outdoor exposure, which showed a protective effect. Likewise, socioeconomic variables such as maternal and paternal unemployment were associated with greater refractive change toward myopia. Sex differences were observed in myopia incidence (higher in females than males), whereas near-work exposure was not analysed for its influence on myopia incidence; therefore, no conclusions can be drawn regarding its effect on myopia development in this cohort.

## Supplementary information

Supplementary Information

## Data Availability

The data presented in this study are available from the corresponding author upon reasonable request, due to ethical restrictions.
