# Supplementary material for: Incidence and Risk Factors of Refractive Error in Children in Spain: CISViT Project
Source: Ophthalmic Physiol Opt. 2026 May 5;46(3):671–80. doi: 10.1007/s44402-026-00086-4 (PMC13369020; doi:10.1007/s44402-026-00086-4)
Supplement: Supplementary file 1 — Supplementary Information [file 44402_2026_86_MOESM1_ESM.pdf]

QÜESTIONARI FAMÍLIA

Num Openvisió

Nom nen/a:	Data naixement:
Escola:	
Ètnia: Caucàsic Amèrica llatina Oriental Hindú Altres____________________________	

Història mèdica del nen/a:

- **Edat gestacional (en setmanes)** ....
- **Pes en néixer (en grams)** ....
- **Pes i talla actual** ....

Descripció de la unitat familiar

	Universitaris o superiors	Secundaris / Formació professional	Primaris	Sense estudis
**Nivell d'estudis del pare**				
**Nivell d'estudis de la mare**				

	Treballa (o en situació de baixa laboral)	No treballa (aturat/da)	Altres (feines de la llar, estudiant...)
**Situació laboral del pare**			
**Situació laboral de la mare**			

	Sí, menor de -6,00D	Sí, igual o superior a -6,00D	No, no és miop	Operat de miopia	Edat d'aparició
**El pare és miop?**					
**La mare és miop?**					

Descripció del nen/a

	Sí, atropina	Sí, Orto-K	Sí, LC toves de control de miopia	No, cap tractament de control de miopia
**Si el nen/a és miop,** ha fet algún tractament pel **control** de la miopia?				

Indica el número d'hores que el nen/a dedica **al dia entre setmana, més enllà de les que passa a classe, a...**	
**Fer activitats a l'aire lliure: Esports, jocs al parc, etc...**	
**Tasques en visió propera: Llegir, escriure, Tauletes digitals, Ordinador, etc...**	

Hores de son que el nen/a dorm **al dia entre setmana**:	
